# Construction of Human Periodontal Ligament Constitutive Model Based on Collagen Fiber Content

**DOI:** 10.3390/ma16196582

**Published:** 2023-10-06

**Authors:** Bin Wu, Na Li, Mao Liu, Ke Cheng, Di Jiang, Yang Yi, Songyun Ma, Bin Yan, Yi Lu

**Affiliations:** 1 College of Mechanical and Electronic Engineering, Nanjing Forestry University, Nanjing 210037, China; wubin@njfu.edu.cn (B.W.); li1209486235@163.com (N.L.); jiangdi_jd@163.com (D.J.); 230169307@seu.edu.cn (Y.Y.); 2 Department of Orthodontics, Affiliated Hospital of Stomatology, Nanjing Medical University, Nanjing 210029, China; maoliu@stu.njmu.edu.cn; 3 Jiangsu Province Key Laboratory of Oral Diseases, Nanjing 210029, China; 4 Jiangsu Province Engineering Research Center of Stomatological Translational Medicine, Nanjing 210029, China; 5 College of Mechanical Engineering, Southeast University, Nanjing 210018, China; 18251982081@163.com; 6 Institute of General Mechanics, RWTH-Aachen University, 52062 Aachen, Germany; ma@iam.rwth-aachen.de

**Keywords:** collagen fiber content, constitutive model, human periodontal ligament, nano-indentation experiment, viscoelasticity

## Abstract

Periodontal ligament (PDL) is mainly composed of collagen fiber bundles, and the content of collagen fiber is an important factor affecting the mechanical properties of PDL. Based on this, the purpose of this study is to explore the effect of the PDL collagen fiber content on its viscoelastic mechanical behavior. Transverse and longitudinal samples of different regions of PDL were obtained from the human maxilla. The fiber content at different regions of human PDL was quantitatively measured using image processing software, and a new viscoelastic constitutive model was constructed based on the fiber content. The nano-indentation experiment was carried out with a loading rate of 0.5 mN·s^−1^, a peak load of 3 mN, and a holding time of 200 s, and the model parameters were obtained through the experiment data. The results showed that with the increase of fiber content, the deformation resistance of PDL also increased, and compared with the neck and middle region, the compressive strain in the apical region of PDL was the largest. The range of reduced elastic modulus of human PDL was calculated to be 0.39~5.08 MPa. The results of the experimental data and the viscoelastic constitutive model fit well, indicating that the model can well describe the viscoelastic behavior of human PDL.

## 1. Introduction

Periodontal ligament (PDL) is a dense connective tissue surrounding the dentin and connecting the dentin and alveolar bone [1,2]. During orthodontic treatment, the PDL will respond to the orthodontic force and act as a medium to transmit the external force to the alveolar bone, eventually triggering the remodeling of the alveolar bone to move the teeth [3,4,5,6,7]. PDL is mainly composed of collagen fiber bundles (type I, III, and V collagen), matrix, blood vessels, cells, and so on [8,9]. Among them, collagen fiber bundles are the most abundant component in PDL, accounting for about 50–75% of PDL volume. Therefore, the mechanical behavior of PDL depends largely on the mechanical properties of PDL fibers. Also, revealing the content of PDL collagen fibers is of great significance for understanding the biomechanical properties of PDL [10,11,12,13,14].

PDL has obvious nonlinear viscoelastic characteristics, and collagen fiber is one of the most important factors affecting its viscoelasticity [15]. Many researchers have studied the viscoelastic behavior of human and animal PDL. Zhou et al. [16] and Tanaka et al. [17] proposed a viscoelastic model for the mechanical experiment of porcine PDL, which proved that PDL is a viscoelastic fluid material under a dynamic shear state. Natali et al. [18] proposed a nonlinear viscoelastic constitutive model to describe the relaxation phenomenon of PDL. Najafidoust et al. [19] performed a compression relaxation experiment on bovine PDL and compared it with a tensile experiment to prove its nonlinear viscoelastic properties during compression. Gupta et al. [20] found that the stress mode of tooth movement was related to the stress distribution of PDL under loading conditions through three-dimensional finite element modeling. Wu et al. [21,22] studied the viscoelasticity of PDL using dynamic mechanical experiments and proved that the viscoelasticity of PDL is related to the frequency.

At present, most studies on human and animal PDL do not consider the effect of collagen fiber content on its viscoelastic behavior. However, the composition and planes of real PDL in different regions from different individuals are different, and the fiber content is also different. The uniaxial tensile and compression tests of bovine PDL, by Pini et al., found that PDL has nonlinear and time-dependent mechanical behavior, and the density and orientation of collagen fibers are the reasons that affect the mechanical properties of PDL [23]. Uhlir et al. [24] used a dynamic mechanical analyzer to perform uniaxial tensile experiments on bovine PDL at different regions. The results showed that the mechanical properties of PDL in different regions were different. Oskui et al. [25] found that dynamic loading rate, loading force amplitude, and fiber content had a certain influence on the viscoelastic properties of bovine PDL by using a dynamic tensile experiment of bovine PDL. Some scholars have conducted mechanical experiments on rat PDL. They found that the density of collagen fibers in different regions of rat PDL is different and revealed the regional inhomogeneity of viscoelastic mechanical behavior of mouse PDL [26]. Other scholars have found that the difference in mechanical properties mainly depended on the structure of collagen fibers in PDL [27,28]. Shingo Hirashima et al. [29] utilized focused ion beam/scanning electron microscopy (FIB/SEM) to observe the three-dimensional ultrastructure of collagen bundles and quantitatively analyzed their histomorphometry. It was found that the strength of the collagen bundles in the horizontal fiber area was higher. Zhong [30] studied the microstructure heterogeneity and biomechanical significance of PDL fibers during loading, quantified the fiber network level, and showed that the structure of PDL collagen fibers was three-dimensional. However, this study only discussed the structure of PDL fibers and did not analyze the effect of the content of collagen fibers. Wu et al. [31] quantified the effect of collagen fiber distribution on the hyperelasticity of human PDL. The spatial angle of collagen fiber was observed by an optical microscope, and the PDL hyperelastic model was constructed based on the observation results. However, the effect of the collagen fiber content on the viscoelastic mechanical properties of PDL was not considered.

The content of collagen fibers in different regions of PDL is different. Combined with mechanical knowledge, it can be speculated that the higher the content of collagen fibers, the stronger the anti-deformation ability of PDL. In order to reveal the effect of the collagen fiber content on the mechanical behavior of PDL, a viscoelastic constitutive model based on collagen fiber content is proposed in this paper. The PDL samples were extracted from fresh corpse jaws, PDL fibers were segmented using image processing software, and the collagen fiber content was measured. The nano-indentation test of human PDL was carried out, and the constitutive model and conjecture proposed in this paper were verified by the experimental data.

## 2. Materials and Methods

### 2.1. Sample Preparation

**Figure 1:**
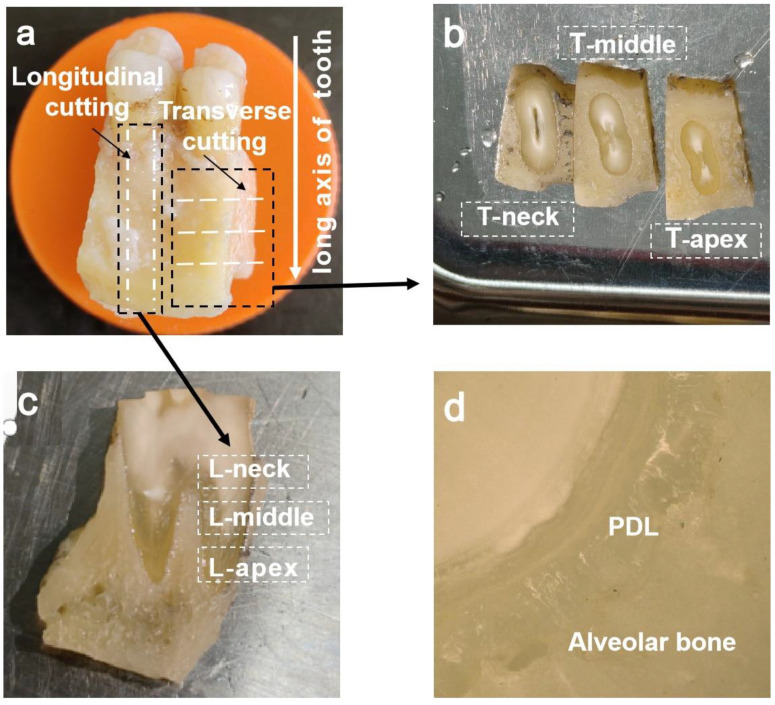
Sample preparation of periodontal ligament (PDL): (**a**) transverse and longitudinal sampling directions; (**b**) the transverse samples; (**c**) the longitudinal samples; and (**d**) the PDL image under the ultra-depth of field microscope.

This study was reviewed and approved (No. (2020)234) by the Institutional Review Board (IRB) of Nanjing Medical University (Nanjing, China). The samples in this paper were taken from two premolars of the human maxilla, which were provided by Nanjing Medical University (Nanjing, China). Human tooth samples were cut with a bone saw to remove the surface soft tissue. As shown in Figure 1a, PDL could be divided into three regions along the long axis of the tooth: neck region, middle region, and apical region. The vertical white dotted lines and horizontal white dashed lines represent the longitudinal (L) sampling direction along the long axis of the tooth and the transverse (T) sampling direction perpendicular to the long axis of the tooth, respectively. The samples of the transverse planes and the longitudinal planes were from two teeth, respectively. The crown was removed using a low-speed cutting machine (500 r/min, Isomet, Buehler, Lake Bluff, IL, USA). The alveolar bone-PDL-dentin samples at different root regions were obtained along the long axis of the tooth and perpendicular to the long axis of the tooth, respectively. The thickness is 2 mm, as shown in Figure 1b,c. The PDL photo under the ultra-depth of field microscope is shown in Figure 1d, and a total of 6 transverse and longitudinal samples of different regions of PDL were obtained, as shown in Table 1. In order to ensure the accuracy of the experiment, the samples were polished before the experiment. After cutting, the samples were immersed in 0.9% normal saline and stored at −20 °C.

### 2.2. Nano-Indentation Experiment

The instrument used in this experiment is the Nano Indenter G200 nano-indentation instrument (Agilent, Santa Clara, CA, USA) for indentation experiments. Before the experiment, the samples were taken out of the freezer and thawed in a water bath at 37 °C. In order to prevent the sample from moving during the experiment, the samples were placed in a fixed bottom column and bonded. In this experiment, a cylindrical indenter was selected, and the radius (R) of the indenter was 100 μm. The areas with PDL thickness greater than 0.2 mm were found under the microscope for experimenting, and five experiment points were selected for each region.

**Figure 2:**
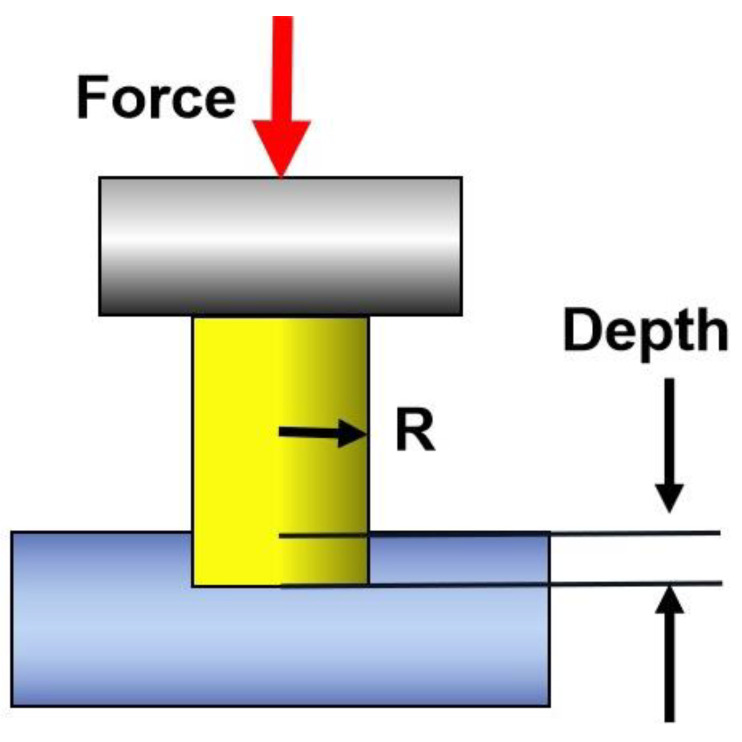
The diagram of cylindrical indenter. Depth is the indentation depth.

The schematic diagram of the nano-indentation mechanical experiment of the cylindrical indenter is shown in Figure 2. The loading rate of the experiment was set to 0.5 mN·s^−1^, the peak load was 3 mN, and the holding time was 200 s [32]. Then, unloading was performed. Finally, the obtained data were derived to obtain the load-indentation depth curves and the displacement-time curves. The displacement-time curves were divided by the PDL thickness to obtain the PDL strain-time curves.

### 2.3. Fiber Content Measurement

A single rooted tooth was isolated from the human jawbone to make specimens, and the surface soft tissue was removed. After fixation, tissue decalcification, dehydration, embedding, and other treatments, the tooth was sliced along the long axis of the tooth. The thickness of the slice was 4 μm, and five slices were evenly selected for Masson staining. The Masson staining sections were quantitatively analyzed under an electron microscope to determine the collagen content of PDL.

**Figure 3:**
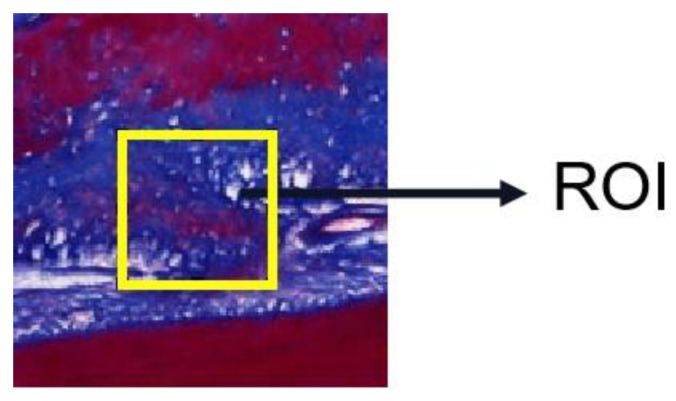
Select the region of interest (ROI).

Image J (National Institutes of Health, Bethesda, MD, USA) was used to select the standardized regions of interest (ROI) in three different regions of PDL for the collagen fiber content measurement, as shown in Figure 3. ROI areas were selected at uniform intervals to measure the collagen content, and the measured values were averaged. The results were expressed as the percentage of Masson stained tissue area to the selected ROI area. The five slices were measured under the same condition, and the collagen fiber content in different regions was averaged to obtain the final fiber content. Collagen fiber content (*V_f_*) is defined by: collagen fiber content *V_f_* (%) = collagen area (px)·[ROI area (px)]^−1^ × 100.

### 2.4. Viscoelastic Constitutive Model Based on Collagen Fiber Content

Under the action of orthodontic force, PDL exhibits viscoelastic creep characteristics, and its response is expressed by Stieltjes integral:

(1) $$\varepsilon (t)=\int_{-\infty }^{t}D(t-\tau )\frac{\partial \sigma (\tau )}{\partial \tau }d\tau$$

Here, *ε*, *σ*, and *D*(*t*) are the strain tensor, stress tensor, and creep tensor, respectively. PDL is a transversely isotropic material under the effect of its internal collagen fibers. At the same time, according to the experimental conditions, the applied load is a step load, that is, *σ*(*t*) = Δ(*t*)·*σ*_0_, *t* ≦ 0, *ε*(*t*) = 0. The Formula (2) can be simplified as follows:

(2) $$\varepsilon \left(t\right)=D\left(t\right)\sigma_{0}$$

where *σ*_0_ can be obtained by *σ*_0_ = *F*·*S*^−1^, *F* is the load, and *S* is the effective section area of PDL.

According to the microscopic observation results, it can be seen that the spatial relationship between collagen fibers and the matrix in PDL is a parallel connection, in which the mechanical behavior of collagen fibers in the holding stage is linear, while the matrix is a viscoelastic fluid material. Therefore, according to the PDL’s mechanical properties, and the spatial relationship between collagen fibers and matrix, the internal components of PDL and its stress state can be expressed as follows:

(3) $$\left\{\begin{array}{l} \overset{•}{\varepsilon }=\frac{{\overset{•}{\sigma }}_{m}}{E_{m}}+\frac{\sigma_{m}}{\eta }\\ \sigma =\sigma_{tf}+\sigma_{m}\\ \varepsilon =\frac{\sigma_{tf}}{E_{tf}} \end{array}\right.$$

The Laplace transform of the above differential equation and the elimination of the intermediate variables (*σ_tf_* and *σ_m_*) can be obtained:

(4) $$\varepsilon^{\prime }\left(s\right)=\left(\frac{\eta s+E_{m}}{(E_{tf}+E_{m})\eta s+E_{tf}E_{m}}\right)\cdot \sigma^{\prime }\left(s\right)$$

where *ε’* and *σ’* are the image functions of *ε* and *σ*, respectively. After Laplace^−1^ transform and arrangement of Equation (4), we obtain:

(5) $$\varepsilon \left(t\right)=\left(\frac{1}{E_{tf}}+\frac{E_{m}}{E_{tf}\left(E_{tf}+E_{m}\right)}\right)e^{-\frac{E_{tf}E_{m}t}{\eta \left(E_{tf}+E_{m}\right)}}\cdot \sigma_{0}$$

(6) $$D\left(t\right)=\frac{1}{E_{tf}}+\frac{E_{m}}{E_{tf}\left(E_{tf}+E_{m}\right)}e^{-\frac{E_{tf}E_{m}t}{\eta \left(E_{tf}+E_{m}\right)}}$$

Formula (6) shows the viscoelastic mechanical properties of PDL under the spatial relationship between the collagen fibers and matrix. In order to further study the effect of the PDL collagen fiber content on its mechanical properties, the Schapery model [33] was introduced:

(7) $$\varepsilon \left(t\right)=g_{0}D_{0}’\sigma +g_{1}\int_{0}^{t}\Delta D’\left(\psi -\psi ’\right)\frac{\partial g_{2}\sigma \left(\tau \right)}{\partial \tau }d\tau$$

where *g*_0_ and *g*_2_ are viscoelastic parameters related to collagen fiber content, and *ψ* and *ψ*′ are the reduction times. *g*_1_ is a parameter related to material strain, which is defined as:

(8) $$g_{1}=\frac{\Delta \varepsilon_{c}-\varepsilon_{p}}{\Delta \varepsilon_{c}-\varepsilon_{0}-\varepsilon_{p}},\varepsilon_{0}=\varepsilon_{b0}-\varepsilon_{r0}$$

Among them, Δ*ε_c_*, *ε_p_*, *ε_b_*_0_, and *ε_r_*_0_ are creep deformation, plastic deformation, unloading rebound deformation, and loading elastic deformation, respectively. According to the study by Zhang et al. [34], Formula (7) can be expressed as:

(9) $$\varepsilon \left(t\right)=(g_{0}D_{0}’+g_{1}g_{2}\Delta D’\left(\frac{t}{\alpha_{\sigma }}\right))\cdot \sigma$$

where *α_σ_* is a stress-dependent nonlinear factor, and *D*′(*t*) is the creep compliance expression of the k-order generalized Kelvin-Voigt model:

(10) $$D^{\prime }\left(t\right)=D_{0}’+D_{k}’\overset{n}{\underset{k=1}{\sum }}\left(1-e^{-\frac{t}{\tau_{k}}}\right)$$

where *τ_k_* = *η_k_*′·*D_k_*′^−1^. For PDL, creep compliance can be expressed by *D*(*t*) and *D*′(*t*), that is, *D*(*t*) = *D*′(*t*):

(11) $$D_{0}’+\overset{n}{\underset{k=1}{\sum }}D_{k}’\left(1-e^{-\frac{t}{\tau_{k}}}\right)=\left(\frac{1}{E_{tf}}+\frac{E_{m}}{E_{tf}\left(E_{tf}+E_{m}\right)}\right)e^{-\frac{E_{tf}E_{m}t}{\eta \left(E_{tf}+E_{m}\right)}}$$

It is easy to know that *k* = 1, so the relationship between the parameters is:

(12) $$\left\{\begin{array}{l} D_{0}’+D_{1}’=\frac{1}{E_{tf}}\\ -D_{1}’e^{t\frac{1}{\eta_{1}^{’}D_{1}’}}=\frac{E_{2}}{E_{tf}\left(E_{tf}+E_{m}\right)}e^{-t\frac{E_{tf}E_{m}}{\eta \left(E_{tf}+E_{m}\right)}}\\ \frac{1}{\eta_{1}’D_{1}’}=\frac{E_{f}E_{m}}{\eta \left(E_{tf}+E_{m}\right)} \end{array}\right.$$

(13) $$\left\{\begin{array}{l} D_{0}’=\frac{2E_{m}+E_{tf}}{E_{tf}\left(E_{tf}+E_{m}\right)}\\ D_{1}’=-\frac{E_{m}}{E_{tf}\left(E_{tf}+E_{m}\right)}\\ \eta_{1}’=-\frac{\eta {\left(E_{tf}+E_{m}\right)}^{2}}{E_{m}^{2}} \end{array}\right.$$

Substituting Equation (13) into Equation (9), the PDL viscoelastic constitutive model representing the internal collagen fiber content and the spatial relationship between the fiber and the matrix can be obtained:

(14) $$\varepsilon \left(t\right)=g_{0}\frac{2E_{m}+E_{tf}}{E_{tf}\left(E_{tf}+E_{m}\right)}\sigma_{0}-g_{1}g_{2}\frac{E_{m}}{E_{tf}\left(E_{tf}+E_{m}\right)}\left[1-e^{-t\frac{E_{tf}E_{m}}{\eta \left(E_{tf}+E_{m}\right)}}\right]\cdot \sigma_{0}$$

## 3. Results

### 3.1. Analysis of Experimental Data Results

**Figure 4:**
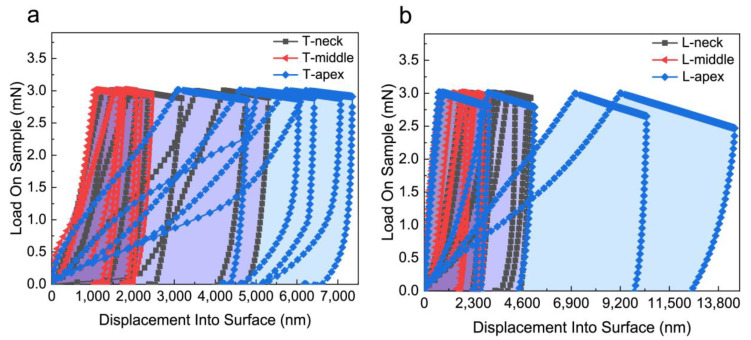
Load-indentation depth curves of five points in the apical, middle, and neck regions in different planes of PDL: (**a**) the samples come from the transverse plane; (**b**) the samples come from the longitudinal plane.

**Figure 5:**
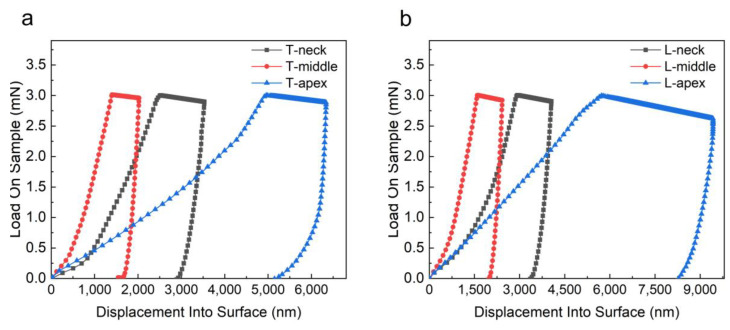
The average load-indentation depth curves of the apical, middle, and neck regions of the PDL in different planes: (**a**) the samples come from the transverse plane; (**b**) the samples come from the longitudinal plane.

**Figure 6:**
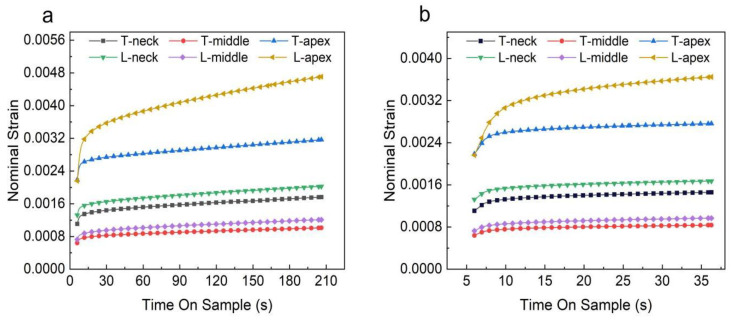
The strain-time curves of PDL in the apical, middle, and neck regions in different planes: (**a**) the strain-time curves of PDL in different regions of different planes during the whole holding stage; (**b**) the strain-time curves of PDL in different regions of different planes in the first 30 s.

The load-depth curves of five points in different regions of PDL obtained from the experimental data are shown in Figure 4. According to the results of Figure 4, it could be seen that the PDL curve distribution in the middle region was more concentrated, the distribution area was the smallest, and the curve shape was more consistent, while the PDL curves in the neck region and the apical region were more dispersed. The difference between the PDL curves at different points in the apical region of the root was large, and the distribution area was the largest. The trend of the transverse samples and the longitudinal samples was consistent, and the indentation depths of the longitudinal section PDL samples were generally greater than those of the transverse samples. In order to facilitate the observation and eliminate the errors, the load-displacement data obtained from the points of each region was averaged and plotted. The results shown in Figure 5 show that the deformation of the PDL in the middle region during the loading stage and the indentation depth during the holding stage were smaller than those of the PDL in the neck region and apical region, and the results of the transverse and longitudinal samples were consistent. The strain-time curves obtained by the nano-indentation experiment are shown in Figure 6. According to the curves, the strain change of the PDL in the apical region with time was significantly greater than that of the middle region and neck region. In the first 30 s, the strain of each sample increased obviously with time (Figure 6b), and the strain increased slowly with time after 100 s (Figure 6a).

The Oliver–Pharr method was used to calculate the reduced elastic modulus (E*) of PDL in different regions of the curve unloading section, and the calculation results are shown in Table 2. From the table, the reduced elastic modulus of human PDL ranged from 0.39 to 5.08 MPa. The reduced elastic modulus of PDL in the middle of the root was greater than that in the neck region, the reduced elastic modulus of PDL in the neck region was greater than that in the apical, and the reduced elastic modulus of PDL samples in the transverse plane was greater than that in the longitudinal plane.

### 3.2. Fiber Content Measurement Results

The fiber content in the apical, middle, and neck regions of human PDL was measured, as shown in Table 3. The results showed that there were some differences in fiber content in different regions of human PDL. The content of PDL collagen fibers in the apical region was about 52%, in the middle region was about 63.1%, and in the neck region was about 60.3%. The content of PDL fiber in the middle region was higher, and the content of fiber in the apical region was relatively lower.

### 3.3. Fitting Results

**Figure 7:**
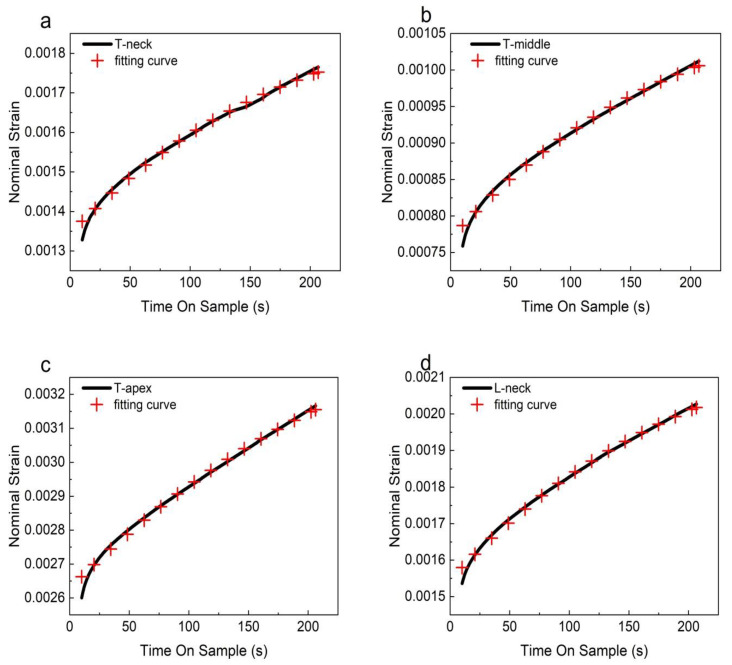

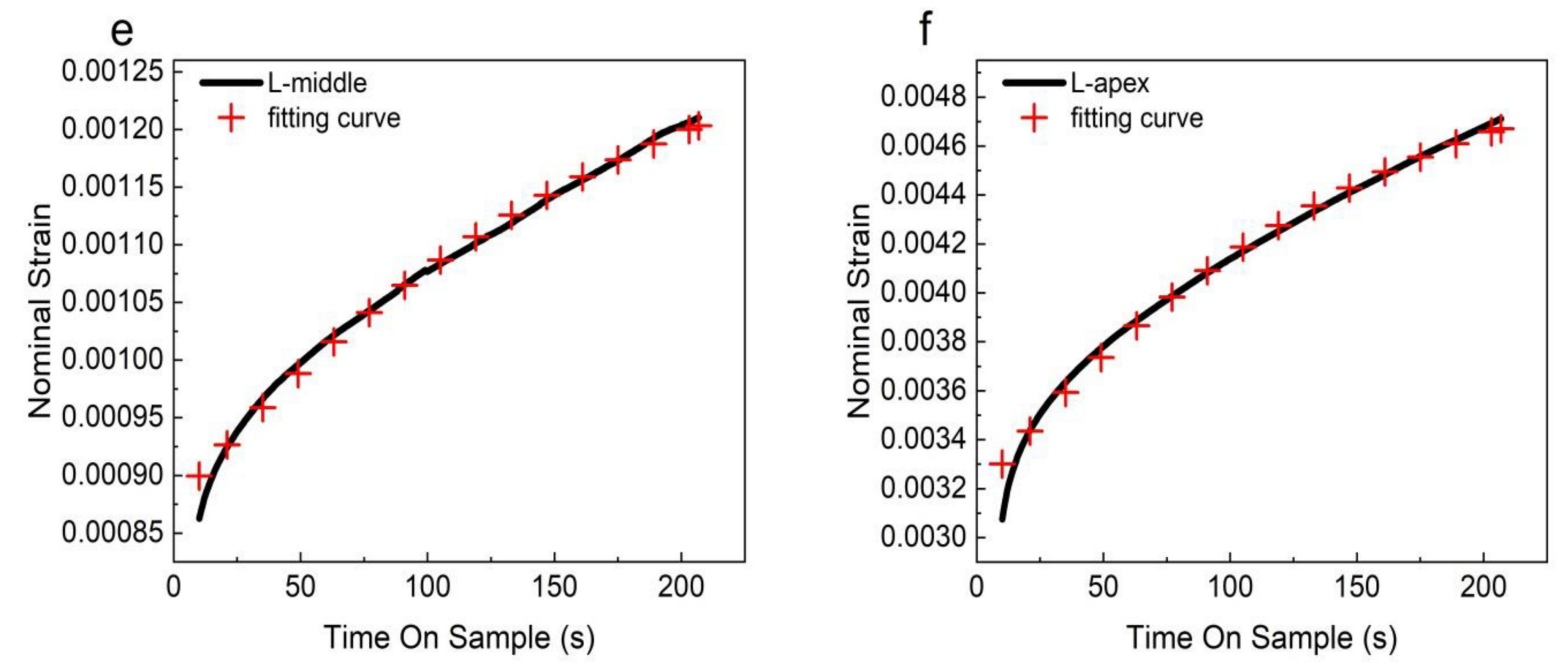
Comparison of strain-time curves and fitting results of PDL samples in transverse and longitudinal planes: (**a**–**c**) are the PDL samples of the neck region, middle region, and apical region of the transverse plane; (**d**–**f**) are the PDL samples of the neck region, middle region, and apical region at the longitudinal plane.

For nonlinear viscoelastic materials, according to previous studies, PDL strain can recover the original length of deformation due to its rebound before exceeding its fiber damage threshold. According to Formula (8), *ε*_0_ = 0 can be obtained, that is, *g*_1_ = 1. Therefore, the parameters *g*_0_, *g*_2_, and *η* can be obtained by fitting the experimental data with Formula (14), and the PDL creep–strain-time relationship, namely the PDL viscoelastic constitutive equation, is finally obtained. The fitting results are shown in Figure 7. The parameters of the PDL viscoelastic model are shown in Table 4.

## 4. Discussion

According to the results of the nano-indentation experiment, the indentation depth of PDL samples in different regions of humans increased to different degrees in the holding stage. With the increase in time, the indentation depth of each sample in the holding stage gradually increased, and PDL showed time-related viscoelastic characteristics. The structure of animal and human PDL is similar, but due to the different biological diversity and living habits, the viscoelastic properties of human and animal PDL will be different. Therefore, the mechanical properties of animal tissues cannot completely replace human tissues. Therefore, in order to more accurately reflect the tissue characteristics of human PDL, the samples used in this study were taken from the human maxilla.

The measurement results of the PDL collagen fiber content showed that the collagen fiber content of human PDL accounted for about 50–70% of the PDL volume, and there were some differences in fiber content in different regions. On the one hand, from the results of the nano-indentation experiment, the strain of PDL in the middle region was the smallest under step load, and the strain of PDL in the apical region was the largest, which was caused by the content of collagen fibers and the spatial structure of PDL. PDL collagen fibers have a certain preferred direction in different root regions. The content of PDL collagen fibers in the middle of the root is the highest, which mainly plays a role in the process of chewing and tooth occlusion. It is distributed at an oblique angle of 45° and is interwoven, which increases the stiffness of the fibers to a certain extent [26,35]. The content of PDL collagen fibers in the apical region is low, and the fibers are radially distributed to connect the alveolar bone and teeth. Therefore, under the same compressive load, the angle between the spatial direction of PDL collagen fibers and the direction of force is large in the apical region, and the solid content of PDL in the apical region is small and the volume of liquid is large. The ability of PDL to withstand load is weaker and the compressive strength is the worst, which is consistent with the research results of Qian et al. [36].

**Figure 8:**
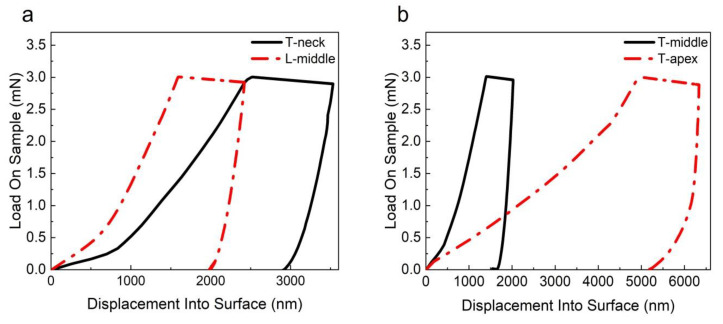
The comparison results of samples from transverse and longitudinal planes: (**a**) the load-depth curve of PDL in the neck region of the transverse plane and PDL in the middle region of the longitudinal plane; (**b**) the load-depth curve of PDL in the middle region and the apical region of the transverse plane.

On the other hand, According to the study of Wu et al. [31], it could be seen that the force direction of the PDL fibers in the neck region is consistent with that of the PDL fibers in the middle region on the longitudinal plane, and the force direction of the PDL fibers in the middle region and the apical region is consistent on the transverse plane. Therefore, in order to reduce the influence of the spatial distribution of PDL on its mechanical properties, the experiment results of the transverse and longitudinal samples were compared, and the results are shown in Figure 8. The results show that when the spatial angle of the fiber was similar, the loading deformation and indentation depth of the PDL at the transverse plane of the neck region were greater than those of the PDL at the longitudinal plane of the middle region; the loading deformation and indentation depth of the PDL at the apical region of the transverse plane were also greater than those of the middle region.

**Figure 9:**
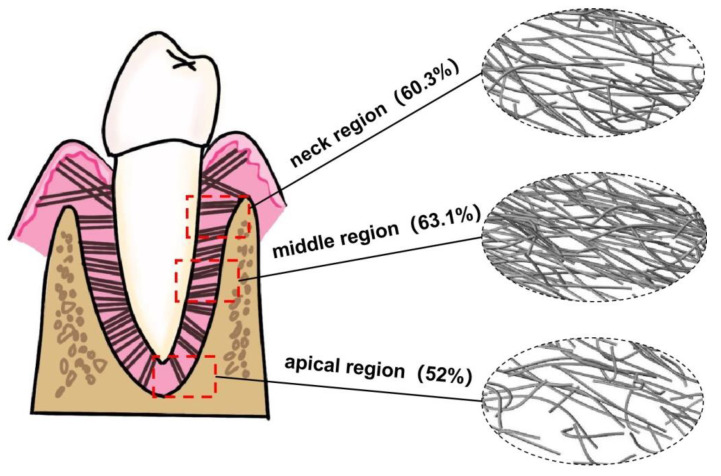
The schematic diagram of deformation resistance of PDL.

In addition, PDL is an anisotropic material. The spatial angle, arrangement distribution, and preferred direction of collagen fibers in different planes will affect the mechanical properties of PDL, which is one of the factors that lead to why the creep deformation of PDL in the longitudinal plane is significantly greater than that in the transverse plane. Simultaneously, the force-bearing of muscle and chewing habits of individuals of different ages are different, and the main functions of different teeth of the same individual are different. These factors will also lead to certain differences in the mechanical properties of PDL. Combined with the comparative experiment of the transverse plane and longitudinal plane and mechanical knowledge, it is speculated that the content of collagen fiber is an important factor in the mechanical properties of PDL. The higher the content of the PDL fiber is, the more the solid component is and the stronger the deformation resistance is. Otherwise, the deformation resistance is weaker, which is consistent with the previous hypothesis. The schematic diagram is shown in Figure 9.

PDL contains liquid, so PDL is usually considered an incompressible material. However, from a mechanical point of view, both the solid and liquid components of PDL have a certain influence on its mechanical properties [37]. The recent research has shown that the experimental data of viscoelastic fluid in PDL were more inclined to the solid viscoelastic properties of collagen fibers [16], that is, PDL collagen fibers play a greater role in the transmission of orthodontic force. In addition, due to the small size and complex structure of PDL, the load transfer mechanism between the microstructure and the fibers is not clear. Therefore, the next stage will consider the liquid–solid coupling effect of the fibers and matrix, the structure of the fibers, and the interaction force between the fibers and the fibers so as to construct a constitutive model that can more accurately describe the mechanical properties of PDL at the macro and micro levels.

The current experimental research has certain limitations and needs further study. First of all, due to the limitation of test technology and the complexity of the internal components of the sample, the direction of collagen fibers is not considered in the nano-indentation experiment, and further experimental experiments (such as strict control of the direction of the PDL collagen fiber group) are needed to construct more accurate constitutive parameters. Secondly, although the proposed viscoelastic model can accurately predict the biomechanical behavior of PDL, this paper regards PDL as a transversely isotropic material, ignoring the anisotropic mechanical properties of PDL, and the model still needs to be optimized.

## 5. Conclusions

In this study, the fiber content of different layers and different root regions of PDL was studied experimentally, and a new viscoelastic constitutive model was proposed based on the fiber content of PDL. Nano-indentation experiments showed that the higher the content of PDL fibers is, the stronger the anti-deformation ability of PDL is. Also, it was found that the anti-deformation ability of PDL in the middle region was the strongest, the apical region was the opposite, and the anti-deformation ability of PDL in the transverse plane was greater than that in the longitudinal plane. The reduced elastic modulus of human PDL was calculated to be 0.39 ~ 5.08 MPa. The constitutive model established in this study can analyze and predict the deformation of PDL according to the different fiber content.

## Figures and Tables

**Table 1:** The transverse and longitudinal samples in different regions of PDL.

	Transverse Regions	Longitudinal Regions
Neck	T-neck	L-neck
Middle	T-middle	L-middle
Apex	T-apex	L-apex

**Table 2:** Reduced elastic modulus of PDL in different layers and regions.

	Transverse Plane/MPa	Longitudinal Plane/MPa
Neck	4.28	2.98
Middle	5.08	4.79
Apex	1.03	0.39

**Table 3:** The volume fraction of collagen fibers in each slice.

*V_f_ (%)*	Section 1	Section 2	Section 3	Section 4	Section 5	Average
Apex	62.198	59.01	41.2	51.417	46.116	51.9882
Middle	58.249	65.771	65.65	61.369	64.669	63.1416
Neck	60.007	51.991	53.889	67.895	67.78	60.3124

**Table 4:** Creep model parameters of PDL in nano-indentation experiment.

	Neck 1	Neck 2	Middle 1	Middle 2	Apex 1	Apex 2
$g_{0}$	0.0049	0.0057	0.0030	0.0034	0.0081	0.0098
$g_{2}$	−0.0096	−0.0118	−0.0060	−0.0082	−0.0124	−0.0242
η	260.8893	289.5958	264.3402	254.2570	370.6347	191.9794
*R^2^*	0.995	0.997	0.9985	0.995	0.996	0.993

## Data Availability

Not applicable.
